# Validation of the self-reported hearing questions in the Irish Longitudinal Study on Ageing against the Whispered Voice Test

**DOI:** 10.1186/1756-0500-7-361

**Published:** 2014-06-14

**Authors:** William Kenny Gibson, Hilary Cronin, Rose Anne Kenny, Annalisa Setti

**Affiliations:** 1 The Irish Longitudinal Study on Ageing, TILDA, Trinity College Dublin, Chemistry Extension Building, Dublin, Ireland; 2 Mercer’s Institute for Successful Ageing, St. James Hospital, Dublin, Ireland; 3 School of Applied Psychology, University College Cork, Cork, Ireland

**Keywords:** Hearing, Whispered voice test, Self-report, Speech

## Abstract

**Background:**

Self report questions are often used in population studies to assess sensory efficacy and decline. These questions differ in their validity in assessing sensory impairment depending on the wording of the question and the characteristics of the population. We tested the validity of the self-report questions on hearing efficacy (self reported hearing, ability in following a conversation, use of a telephone and use of hearing aids) used in The Irish Longitudinal Study on Ageing (TILDA).

**Methods:**

We tested sensitivity and specificity, positive and negative predictive values of each question against the Whispered Voice Test, a relatively easy to administer and cost effective alternative to the standard audiometric test.

**Results:**

In this population the question ‘Is your hearing (with or without a hearing appliance)/ Excellent/Very Good/Good/Fair/Poor?’ showed the best diagnostic value in relation to the other questions (sensitivity 55.56% and specificity 94.67%). The question ‘Can you use a normal telephone?’ was deemed ineffective because of a very poor sensitivity (5.56%) and was proposed for exclusion from subsequent waves of TILDA.

**Conclusions:**

We showed that this validity check was useful to select the questions that most effectively assess hearing deficits and provided crucial information for the subsequent waves. We argue that longitudinal studies using self-reports of sensory efficacy would benefit from a similar check.

## Background

Hearing loss is one of the most common chronic conditions affecting the ageing population with a reported prevalence of between 20% and 40% among adults aged over 50 [1-3] or even higher (over 60%) when considering speech frequencies among adults aged 70 and over [4]. Relatively few people suffering from hearing loss choose to utilize amplification devices hence leaving their hearing loss uncorrected [5]. The implications of hearing loss stretch beyond a simple decline in sensory function; hearing impairment is physically disabling [6,7] especially when combined with visual impairment [8,9]; it results in various negative outcomes including depression, anxiety and social isolation [10] and it has been associated with poorer cognitive function including memory and executive function [11]. Therefore hearing loss is associated with a diminished quality of life in older adults with hearing loss [12] and their immediate relatives [13].

In large survey studies it is often difficult to introduce objective hearing tests, such as the pure tone audiometric test, due to time constraints, costs and compliance. Therefore self reports are used to assess hearing (e.g. English Longitudinal Study on Ageing, ELSA [14]; the Survey of Health Ageing and Retirement in Europe, SHARE [15]; and the Longitudinal Aging Study Amsterdam, LASA [16]).

Although self reports of hearing impairment have proven effective in terms of predicting the negative outcomes of hearing loss (e.g. [17]), the extent to which such tests can accurately assess hearing impairment compared with objective hearing measures is not entirely clear [18]. Other factors contribute to the determination of self reported hearing loss such as cognitive abilities, education and individual dispositions [19]. This is captured by the variability in sensitivity and specificity levels found when comparing self rated hearing loss with objective measures between different studies. Differences in wording of questions or criterion used to define hearing loss are also sources of variability (e.g. [20,21]). Therefore to better understand to what extent self reports of hearing loss reflect objective deficits, a direct comparison between tests is needed [22]. This is necessary because differences in population characteristics in terms of culture, education, cognitive status etc. within and across studies may influence the relationship between self reported and objective measures of hearing [19]. This in turn determines whether self reported hearing can be used to study the impact of poor hearing on other functional or cognitive abilities (see e.g. [23]). In other words, to inform comparability it is necessary to determine to what extent self-reports address the sensory deficit they aim to assess.

The primary objective of this study was to examine the accuracy of different self reported hearing loss questions relative to a more objective hearing test, the Whispered Voice Test, when assessing hearing in a population study of ageing in Ireland, the Irish longitudinal study on Ageing (TILDA). Several studies have shown the Whispered Voice Test to be one of the best simple tests in identifying hearing impairment with respect to sensitivity and specificity when compared with audiometric testing [24,25] even if the lack of standardization constitutes a limitation for this test [26]. It should be also noted that the Whispered Voice Test assesses hearing loss in a smaller range of frequencies relative to an audiometric test [25]. Nonetheless the Whispered Voice Test presents the advantage of plausibly leading to higher compliance and lower selection bias than the standard audiometric test; the test is also relatively easy and cost effective to administer. Importantly, it has been shown that this test has a sensitivity of 80–100% and specificity of 80–89% by comparison with pure-tone audiometry in detecting hearing loss in the range 30–40 db loss (range observed in screening criteria for mild to moderate hearing loss assessed by pure tone audiometry (see [24]).

For the purpose of this study, data from the pilot wave of TILDA were analysed which included both subjective and objective measures of hearing loss. We tested the specificity and sensitivity of a series of self rated questions used in TILDA in relation to the Whispered Voice Test in order to determine what questions were most effective in detecting hearing deficits for the purpose of inclusion/exclusion from the subsequent main waves.

## Methods

### Sample

The sample studied here was derived from the second pilot of TILDA which was conducted between 2009 and 2010 and included 291 individuals. There is no enumeration of individuals in Ireland that could be used as a sampling frame, however a list of household addresses was available, and so sampling was initially conducted at the household level. The sample for this pilot study was selected using a RANSAM sampling system [27]. The sampling frame on which this system is based is the Irish Geodirectory, a comprehensive and up-to-date listing and mapping of all residential addresses in the Republic of Ireland compiled by Ordnance Survey Ireland. The target area for the sample in Pilot II comprised Dublin City and the county of Dun Laoghaire/Rathdown. Seven hundred and sixty addresses were randomly selected within this area and each address was visited by a fieldworker. One (randomly selected) household member aged 50 or over was selected as primary respondent for the survey with this person’s spouse (of any age) also selected for interview. Ethical approval was obtained from the Trinity College Dublin research ethics committee, and all participants provided written informed consent.

### Questionnaire

Initially each respondent participated in a computer based questionnaire in their home which was carried out using Computer Assisted Personal Interview (CAPI). This questionnaire covered a broad range of categories from various aspects of physical health (including sensory abilities among which self-rated hearing questions were administered), functional ability, cognitive and psychological well-being, to socio economic information.

Five hearing related questions with multiple choice answers were included in the questionnaire. Questions were designed to detect a range of functional deficits including self assessed hearing deficit and different aspects of functional use of hearing such as following a conversation and using a telephone. For international comparability these were modelled on self report questions from similar studies such as ELSA, SHARE and LASA. The questions and answers (multiple-choice) are listed below:

1. Do you use any of the following appliances to help with your hearing? Also used in ELSA/HRS/SHARE. Answers: Hearing Aid (all of the time); Hearing Aid (some of the time); Amplifier; None of the above

2. Is your hearing with or without a hearing appliance? Also used in LASA, answers: Excellent; Very Good; Good; Fair; Poor

3. Can you follow a conversation with one person? Also used in LASA, answers: No Difficulty; Some Difficulty; Much Difficulty; No I cannot

4. Can you follow a conversation with four people? Also used in LASA, answers: No Difficulty; Some Difficulty; Much Difficulty; No I cannot

5. Can you use a normal telephone? Also used in LASA, answers: No Difficulty; Some Difficulty; Much Difficulty; No I cannot

### General health assessment

Once each participant had completed the CAPI interview, they were asked to take part in a general health assessment in a separate session at the health assessment unit in Trinity College Dublin. Each assessment was conducted by qualified, specially trained research nurses who followed standard operating procedures for all tests and measurements. The health assessment included a comprehensive cardiovascular, cognitive, anthropometric, gait and balance, grip strength measurements and sensory assessment as well as the Whispering Voice Test. On average each assessment took between two and two and half hours to complete.

### Whispered voice test

In accordance with TILDA protocol, the participant was asked to sit with his/her back to the nurse throughout the test. The nurse stood at a distance of 0.6 m from the participant and each ear was tested separately. During the examination, the non-tested ear was masked by the nurse gently occluding the auditory canal with a finger and rubbing the tragus in a circular motion. Starting with the better functioning ear (as determined by the participant), the nurse whispered a combination of numbers and letters (e.g. 4-K-2) to the participant who had to repeat the combination back to her. The nurse was trained to exhale prior to whispering to ensure that the whispering was as quiet as possible. A maximum of six combinations were whispered to each ear and the respondent was required to repeat 3 sequences for each ear correctly in order to pass. Two research nurses administered the test in this pilot. They were extensively trained to fully adhere to the Standard Operating Procedure protocol in administering and scoring the test.

Analyses were carried out with PASW SPSS 18. The result of the better ear for the Whispered Voice Test was used in the analysis. The sensitivity, specificity and positive and negative predictive values of each question were tested. For the purpose of this analysis the following cut-off points were established for the self report questions: in question 1, a response indicating the use of any hearing appliance resulted in a fail. In question 2 a participant was deemed to have failed if they gave a response of ‘fair’ or ‘poor’. In questions 3; 4 and 5 participants passed the test if they responded with ‘no difficulty’, all other responses were considered a fail. These cut-offs optimised the sensitivity and specificity values in relation to the objective hearing assessment based on Receiving Operator Curve (ROC) analysis.

## Results

Two hundred and ninety one respondents (100%) completed the CAPI questionnaire, 168 (57.7%) participants attended the subsequent health assessment and successfully completed the Whispered Voice Test, 8 respondents (2.75%) were unable to take part or complete the Whispered Voice Test and 115 (39.55%) did not have a health assessment. The majority of participants who were administered the Whispered Voice Test was younger (58% under 65 years of age) and female (56%). Of the 168 who were administered the Whispered Voice Test, 150 respondents (89.3%) passed the test and demonstrated no hearing impairment, and 18 respondents (10.7%) failed (prevalence of hearing impairment = 10.7%). Table 1 shows the number of respondents reporting hearing impairment according to the established cut-offs for each of the self-report questions and the corresponding performance (pass/fail) at the Whispered Voice Test.

**Table 1:** Results of the Whispered Voice Test in relation to each of the questions

**Whispered voice test**	**Q1. Do you use any of the following aids or appliances to help you with your hearing?**		**Q2. Is your hearing (with or without a hearing aid)?**		**Q3. Can you follow a conversation with one person (with or without a hearing aid)?**		**Q4. Can you follow a conversation with four people (with or without a hearing aid)?**		**Q5. Can you use a normal telephone?**	
	**No**	**Yes**	**Exc-VG-G**	**Fair-Poor**	**No diff**	**Difficulty**	**No diff**	**Difficulty**	**No diff**	**Difficulty**
Pass	144	6	142	8	142	8	127	23	149	1
Fail	17	1	8	10	14	4	9	9	17	1

The number of participants reporting good or poor hearing and passing or failing the Whispered Voice Test is reported.

The sensitivity and specificity values as well as the positive and negative predictive values for the self-rated hearing questions were then calculated (Table 2). Sensitivity values relate to the percentage of respondents who reported to have poor hearing and also failed the Whispered Voice Test therefore were correctly identified as hearing impaired by the self report question. The specificity relates to the percentage of respondents who were correctly identified by the self report questions as not having a hearing impairment having passed the Whispered Voice Test. The positive predictive value (PPV) is the probability of a respondent having a hearing loss according to the Whispered Voice Test to also showing a hearing impairment in the self reports. The negative predictive value (NPV) is the probability of a respondent not showing a hearing impairment in the Whispered Voice Test to be also identified as not having a hearing impairment by the self report question. These values were calculated for each of the questions separately. Each question yielded a relatively high specificity value (range 84.67% - 99.3%) while there was a considerably greater amount of variation between sensitivity values (range 5.56% - 55.56%).

**Table 2:** Sensitivity, specificity, PPV and NPV of self-report questions

**Self assessment questions**	**Sensitivity CI (95%)**	**Specificity CI (95%)**	**PPV CI (95%)**	**NPV CI (95%)**	**Response**
Do you use any of the following appliances to help with your hearing?	5.56% (0.11% – 11.01%)	96% (92.86% - 99.14%)	14.29% (4.49% - 24.09%)	89.44% (84.69% – 94.19%)	Hearing Aid*
					Amplifier*
					None
Is your hearing (with or w/out hearing appliance)…	55.56% (32.60% - 78.52%)	94.67% (91.08% - 98.26%)	55.56% (32.60% - 78.52%)	94.67% (91.08% - 98.26%)	Excellent
					Very Good
					Good
					Fair*
					Poor*
Can you follow a conversation with one person? With…	22.22% (9.14% - 45.56%)	94.67% (89.82% - 97.23%)	33.33% (13.86% -61.43%)	91.03% (85.49% - 94.55%)	No Difficulty
					Some Difficulty*
					Much Difficulty*
					No I Cannot*
Can you follow a conversation with four people?	50% (26.9% - 73.1%)	84.67% (78.9% - 90.44%)	28.13% (12.55 - 43.71%)	93.4% (89.2% - 97.56%)	No Difficulty
					Some Difficulty*
					Much Difficulty*
					No I Cannot*
Can you use a normal telephone?	5.56% (0.11% – 11.01%)	99.3% (97.39% - 99.95%)	50%	89.76% (84.87% - 94.91%)	No Difficulty
					Some Difficulty*
					Much Difficulty*
					No I Cannot*

(*) Represent a response considered a fail.

The question, “Is your hearing (with or without a hearing appliance)/Excellent; Very Good; Good; Fair; Poor?,” was the most accurate in detecting hearing loss among participants, with 10 participants self-reporting poor hearing (true positive) out of the 18 who failed the Whispered Voice Test (i.e. 8 false negatives) and only 8 participants out of 150 passing the Whispered Voice Test while self-reporting poor hearing (false positives). This test therefore demonstrated the highest sensitivity and specificity values (55.56% and 94.67% respectively). Accordingly the positive and negative predictive values for this question were relatively high within this set of questions. For the self report question “Do you use any of the following appliances….?,” the false negatives were 17 out of 18 participants failing the Whispered Voice Test and the false positives were 6 out of 150 participants passing the test. Accordingly the sensitivity was low (5.56%) while the specificity was far higher (96%). The PPV was only 14% and the NPV 89%. The question regarding a conversation with one person produced 14 false negatives out of 18 people failing the Whispered Voice Test and 8 false positives out of 150 passing the test. The specificity was high (94.67%), however, the sensitivity was poor (22.22%), as indicated by the greater likelihood of false negatives. Accordingly the PPV was low (33%) and the NPV was high (91%). Sensitivity and specificity values from the question on following a conversation with 4 people were relatively convergent with our findings from ‘Is your hearing (with or without a hearing appliance)/Excellent; Very Good; Good; Fair; Poor?’ (50% and 84.67% respectively). False negatives were 9 out of 18 participants failing the Whispered Voice Test and false positives were 23 out of 150 participants passing the Whispered Voice Test. The sensitivity was higher than for the question on following a conversation with one person (50% vs. 22%), implying that fewer respondents with hearing loss were incorrectly diagnosed as being healthy according to this question. The specificity value was lower than that of the question on conversation with one person implying that a higher proportion of participants without hearing impairment could be misdiagnosed with hearing loss. Accordingly the PPV was lower for this question than for the question on following a conversation with one person (28% vs. 33%). The question “Can you use a normal telephone…?’ showed ceiling effects leading to a relatively large number of false negatives (17) and 1 false positive. The sensitivity value was extremely low (5.56%) and the specificity value was high (99.3%).

## Discussion

Answers to five questions on different aspects of participants’ hearing abilities were compared with the results of the Whispered Voice Test. The Whispered Voice Test was not included in the main waves of TILDA due to time constraints; therefore this pilot study is of relevance in order to understand to what extent the self-report questions included in the main waves capture physiological hearing deficits (in the speech frequency range). The Whispered Voice Test is appropriate to determine hearing loss within the 30–40 dBL range although its use as an objective measure of hearing has limitations relative to the audiometric test especially due to the way it is administered [24,26]. Care was taken in TILDA to minimise variability in administering the test by extensively training professional research nurses to deliver it in a standardised way, however a subjective element in the tone of voice used to pronounce the sequence of items to be repeated back cannot be completely excluded and constitutes a limitation in this study. The best match was obtained with the question “Is your hearing (with or without a hearing appliance)/Excellent; Very Good; Good; Fair; Poor?,” showing an acceptable sensitivity and high specificity levels in relation to the outcome of the Whispered Voice Test. The sensitivity value is lower than the value obtained with other questions such as ‘Do you feel that you have a hearing problem?’ [21,28] possibly because the explicit mention of a ‘problem’ represents an easier way to identify an issue than rating how good is one’s hearing. This question has been introduced in way 2 of TILDA. The self-report of use of hearing appliances showed low diagnostic value as the prevalence of hearing aid use among older persons with hearing loss is disproportionately low [5], in addition it is not clear whether false positives could indicate that the hearing aid provides a good correction.

Two questions enquired about the ability to follow a conversation, either with one or four people. Following a conversation requires cognitive abilities as well as good hearing (see e.g. [29]), this is plausibly the reason why the question on following a conversation with one person showed low sensitivity (22.2%) and PPV (33.3%). Cognitive compensatory mechanisms for hearing loss may be more difficult to display when following a conversation with four people therefore this question shows higher sensitivity than the previous (50% vs. 22%), presumably tapping more on hearing deficits. However the low PPV 28.13% in contrast with the higher sensitivity value implies that this test may be also assessing a range of factors that are related to hearing ability (including for example lower education and poorer cognitive function) not only the physiological deficit.

The efficacy of the question on using a telephone in capturing hearing deficits here is questionable, in fact it is plausible to think that respondents interpreted the question more in relation to the instrumental skills required by the use of the telephone than in relation to hearing if we consider that only 2 people reported difficulties in the use of a telephone, while 12 reported difficulties in following a conversation with one person and 32 with four people (both questions assessing relatively similar use of hearing to the use of a telephone).

Following our investigations, the original question set was revised for use in subsequent TILDA waves. The question, ‘Can you use a normal telephone…?’ was omitted for its lack of sensitivity and substituted with the question, ‘Do you feel you have hearing loss…?’ that was shown in the literature to have both high sensitivity and specificity values. The modifications have been introduced from Wave 2. The question regarding the use of hearing aids, despite showing very low sensitivity values was maintained in order to keep track of respondents using corrections for their deficits.

Limitations of this study are the relatively small sample and the lack of audiometric test to be able to compare the self ratings with both the Whispered Voice Test and the standard audiometric test. In addition, in conducting the Whispered Voice Test wax in the ears temporarily limiting hearing abilities was not checked for. Clearly the self reported measures described here cannot substitute more objective assessments in terms of diagnosis of hearing loss and it should be taken into account that The Whispered Voice Test has limitations as an objective measure of hearing [24].

## Conclusions

Sensory decline, in particular hearing and vision, is linked to decline in functional abilities and cognition e.g. [30], therefore it is crucial for longitudinal studies to capture it. The aim of this study was to assess the validity of the questions regarding hearing used in TILDA -and in other epidemiological studies- in mapping onto a more objective test of hearing, namely the Whispered Voice Test. The scope of this investigation was to inform on the reliability of these questions in reporting a hearing deficit as opposed to other intervening factors that may determine the self-reports (e.g. depression, level of education) and to inform subsequent waves on the most effective questions to be included. The question ‘Is your hearing (with or without a hearing aid) ..Excellent/very good/..’ presented the best mapping onto the objective assessment provided by the Whispered Voice Test but its sensitivity is lower than the values obtained when comparing more objective tests of hearing (e.g. the Whispered Voice Test against the audiometric test, [24]). The questions on following a conversation with one or four people presented a good diagnostic value in terms of hearing deficit but they are clearly related also to other abilities (e.g. cognition) that deserve further investigation. The question ‘Can you use a normal telephone?’ was intended to tap on hearing impairment related to daily life problems, however it led to ceiling effects and was deemed insufficiently sensitive to be included in future waves of TILDA. The question ‘Do you feel you have a hearing problem’ (see [22] for validation of a similar wording) was introduced instead.

In sum the validation of self-reported hearing questions used in population studies is useful in determining whether an actual hearing deficit is captured. The Whispered Voice Tests is a viable instrument to assess the validity of these self report measures when the audiometric test cannot be used because of economic or time constraints (but see [26]). In the present study the validation process of the self reported questions against the Whispered Voice Test brought to optimising the set of questions to be included in the main survey, while offering valuable information on the reliability of these questions in assessing hearing deficits as opposed to other psychological and socio-demographic dimensions.

## Competing interests

The authors have no financial or non-financial competing interests to declare.

## Authors’ contributions

WKG designed the study and conducted the analysis. WKG and AS drafted the manuscript; HC and RAK designed the TILDA pilot II and critically revised the manuscript. All authors read and approved the final manuscript.
